# Intrinsic entropy model for feature selection of scRNA-seq data

**DOI:** 10.1093/jmcb/mjac008

**Published:** 2022-01-31

**Authors:** Lin Li, Hui Tang, Rui Xia, Hao Dai, Rui Liu, Luonan Chen

**Affiliations:** State Key Laboratory of Cell Biology, Shanghai Institute of Biochemistry and Cell Biology, CAS Center for Excellence in Molecular Cell Science, Chinese Academy of Sciences, Shanghai 200031, China; University of Chinese Academy of Sciences, Beijing 100049, China; School of Mathematics, South China University of Technology, Guangzhou 510640, China; State Key Laboratory of Cell Biology, Shanghai Institute of Biochemistry and Cell Biology, CAS Center for Excellence in Molecular Cell Science, Chinese Academy of Sciences, Shanghai 200031, China; University of Chinese Academy of Sciences, Beijing 100049, China; State Key Laboratory of Cell Biology, Shanghai Institute of Biochemistry and Cell Biology, CAS Center for Excellence in Molecular Cell Science, Chinese Academy of Sciences, Shanghai 200031, China; School of Mathematics, South China University of Technology, Guangzhou 510640, China; State Key Laboratory of Cell Biology, Shanghai Institute of Biochemistry and Cell Biology, CAS Center for Excellence in Molecular Cell Science, Chinese Academy of Sciences, Shanghai 200031, China; Center for Excellence in Animal Evolution and Genetics, Chinese Academy of Sciences, Kunming 650223, China; Key Laboratory of Systems Health Science of Zhejiang Province, Hangzhou Institute for Advanced Study, University of Chinese Academy of Sciences, Chinese Academy of Sciences, Hangzhou 310024, China; Guangdong Institute of Intelligence Science and Technology, Zhuhai 519031, China

**Keywords:** scRNA-seq, feature selection, intrinsic entropy, extrinsic entropy, entropy decomposition, informative genes

## Abstract

Recent advances of single-cell RNA sequencing (scRNA-seq) technologies have led to extensive study of cellular heterogeneity and cell-to-cell variation. However, the high frequency of dropout events and noise in scRNA-seq data confounds the accuracy of the downstream analysis, i.e. clustering analysis, whose accuracy depends heavily on the selected feature genes. Here, by deriving an entropy decomposition formula, we propose a feature selection method, i.e. an intrinsic entropy (IE) model, to identify the informative genes for accurately clustering analysis. Specifically, by eliminating the ‘noisy’ fluctuation or extrinsic entropy (EE), we extract the IE of each gene from the total entropy (TE), i.e. TE = IE + EE. We show that the IE of each gene actually reflects the regulatory fluctuation of this gene in a cellular process, and thus high-IE genes provide rich information on cell type or state analysis. To validate the performance of the high-IE genes, we conduct computational analysis on both simulated datasets and real single-cell datasets by comparing with other representative methods. The results show that our IE model is not only broadly applicable and robust for different clustering and classification methods, but also sensitive for novel cell types. Our results also demonstrate that the intrinsic entropy/fluctuation of a gene serves as information rather than noise in contrast to its total entropy/fluctuation.

## Introduction

Single-cell technologies have emerged as a powerful tool to enable the identification and characterization of pure cell types (Kolodziejczyk et al., 2015; Papalexi and Satija, 2018). An exponential increase of the single-cell RNA sequencing (scRNA-seq) datasets presents a computational challenge. Recently, many efficient computational methods have been proposed for accurate classification of scRNA-seq, such as Seurat (Stuart et al., 2019) and SC3 (Kiselev et al., 2017). However, the high frequency of dropout events and noise in scRNA-seq data still confounds clustering analysis. In addition, the ‘curse of dimensionality’ in scRNA-seq data makes it difficult to obtain accurate clusters. Feature selection can identify the most informative genes and then reduce the noise of scRNA-seq data, thus enhancing computational efficiency of dimension reduction and clustering. Recently, diverse methods have been proposed for the selection of informative genes. HVG (Brennecke et al., 2013) can identify the biologically variable genes through a generalized linear model; however, many low-expression genes would often be selected due to the high levels of dropout. GiniClust2 (Tsoucas and Yuan, 2018) could identify the highly variable genes by Gini coefficient and further was specially applied to detect rare cell types. In the S–E model (Liu et al., 2020), the differential entropy was computed to capture the informative genes based on hypothesis testing. However, the S–E model obtained the degree of randomness of genes based on the expression data. The dropout and high noise of scRNA-seq data hinder the obtainment of real fluctuation of genes in a biological process.

Here, we developed a novel feature selection model that can accurately estimate the inherent fluctuation of each gene using the intrinsic entropy (IE) model. Generally, the fluctuation of a random variable in a complex system can be caused by the intrinsic fluctuation generated by its inherent dynamics and the extrinsic randomness influenced by its environment or all other variables. In this work, we decompose the total entropy (TE) or ‘raw’ fluctuation of each variable/gene into its IE and extrinsic entropy (EE) based on information theory. Focusing on a specific variable/gene, we show that IE and EE of this gene actually characterize the inherent fluctuation and ‘noisy’ fluctuation of this gene, respectively. In particular, IE can capture the degree of the regulatory fluctuation on each gene in a cellular process, and thus can be used to identify the informative genes for accurate clustering and classification of cells from scRNA-seq data. We demonstrate that the high-IE genes are highly informative and can significantly improve the performance of clustering in both simulated datasets and real datasets. The computational analysis further shows that the IE model could not only accurately classify the dropout cells but also identify novel cell types. The IE model is implemented in an open-source R package IEntropy (https://github.com/LinLi-0909/IEntropy).

## Results

### Overview of the IE model

**Figure 1:**
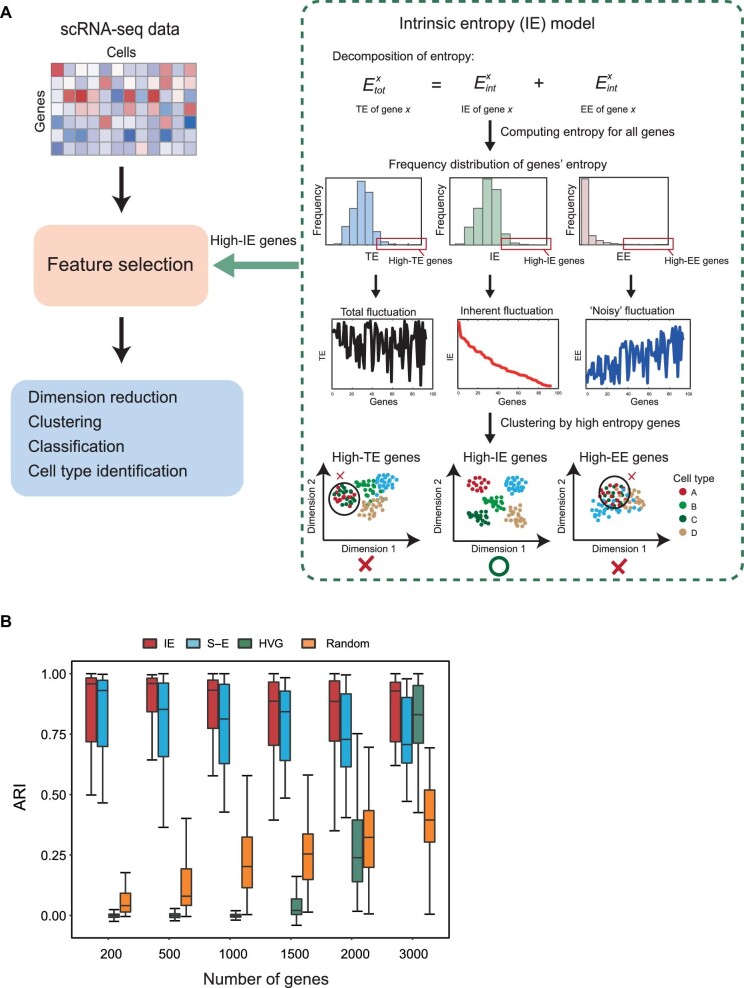
Overview of the IE model. (**A**) TE of each gene can be decomposed into IE and EE in the IE model. High-IE genes are more informative and thus can be used for downstream analysis of scRNA-seq data. (**B**) Performance of various feature selection methods evaluated by ARI in simulated datasets. The IE model shows better performance than other methods in terms of ARI.

In our IE model, the TE of a gene *x* can be decomposed into its IE and EE. Notably, we observed that high-EE genes (i.e. with high EE values) actually fail to distinguish different cell types (Figure 1A; Supplementary Figure S1). In contrast, high-IE genes (i.e. with high IE values) are able to reliably identify different cell types, compared with the genes with high TE or EE. Thus, to capture the real fluctuation generated by the inherent dynamics of each gene, we developed the IE model, with which we can obtain the informative genes by selecting high-IE genes. Any clustering or classification method can be used for further downstream analysis with those high-IE genes.

### The IE model identifies informative genes for accurate clustering

To illustrate the performance of our IE model, we first compared it with current state-of-the-art feature selection methods, i.e. S–E (Liu et al., 2020) and HVG (Brennecke et al., 2013), on simulated datasets. The randomly selected genes were also considered to compare with these three feature selection methods. Specifically, we generated 600 simulated scRNA-seq datasets based on a gamma-Poisson distribution using Splatter (Zappia et al., 2017). The adjusted rand index (ARI) was used to evaluate the performance of these methods. As shown in Figure 1B, clearly our method with high-IE genes was consistently superior to other methods when selecting a different number (200–3000) of genes. When fewer genes were selected, HVG showed poor performance, while S–E performed well. However, S–E did not perform better than the IE model when more genes were used. Furthermore, high-IE genes could be also applied on batch correction of scRNA-seq data (Supplementary Figure S2).

**Figure 2:**
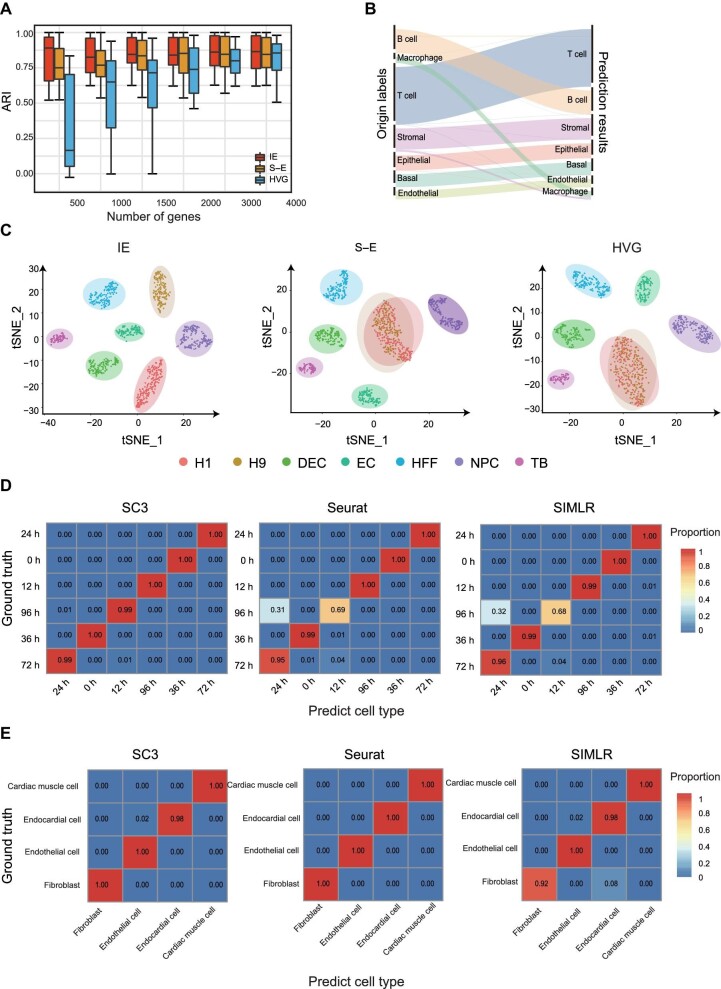
The IE model accurately identifies informative genes. (**A**) Performance of three feature selection methods in real datasets measured by ARI. (**B**) The Sankey diagram shows the clustering result (ARI = 0.97) of the Tabula Muris (Mammary Gland) dataset based on genes selected by the IE model (high-IE genes). (**C**) t-SNE plots show the dimensional reduction results based on the genes selected by the IE model, S–E, and HVG. (**D**) Heatmap plots for the confusion matrix of the results by different clustering methods on the Chu-time dataset. The clustering analysis was performed based on genes selected by the IE model. (**E**) Heatmap plots for the confusion matrix of the results by different clustering methods on the Tabula Muris (Heart and Aorta) dataset. The clustering analysis was performed based on genes selected by the IE model.

Then, to validate the performance of the IE model in real datasets, we considered 14 published scRNA-seq datasets (Supplementary Table S1) to benchmark our model. These datasets were sequenced with droplet-based protocols and full-length protocols (Supplementary Table S1). The accuracy of the clustering can intuitively reflect the performance of feature selection methods (Kiselev et al., 2018), and hence we performed clustering analysis after selecting feature genes. The results validated that high-IE genes (500‒4000) selected by the IE model consistently have better performance in clustering than others (Figure 2A). Overall, these results show that the IE model can reliably identify informative genes, i.e. high-IE genes, for accurately clustering cells.

### Robustness of the IE model in different clustering methods

Applications of feature selection can improve the performance of downstream analyses, such as dimension reduction and clustering. Here, we first used the Mammary Gland dataset, which is composed of diverse cell types, to demonstrate the performance of the IE model. We classified and annotated these cells by Seurat (Stuart et al., 2019). The Sankey diagram (Figure 2B) revealed that cells were correctly identified (ARI = 0.97). In addition, to demonstrate the sensitivity of our model, we considered the Chu-time dataset, which consists of human progenitors. The IE model separated H1 and H9 embryonic stem cells clearly, while HVG and S–E failed to distinguish these two similar cell types (Figure 2C). These results suggest high sensitivity of the IE model.

To test the performance of the IE model in different clustering methods, we applied SIMLR (Wang et al., 2018), SC3 (Kiselev et al., 2017), and Seurat (Stuart et al., 2019), which are popular clustering methods of scRNA-seq, on two datasets. For the full-length-based dataset (Chu-time) and droplet-based dataset (Heart and Aorta), the confusion matrices of classification showed that the IE model (high-IE genes) performed well in three clustering methods (Figure 2D and E).

### Robustness of the IE model in different classification methods

**Figure 3:**
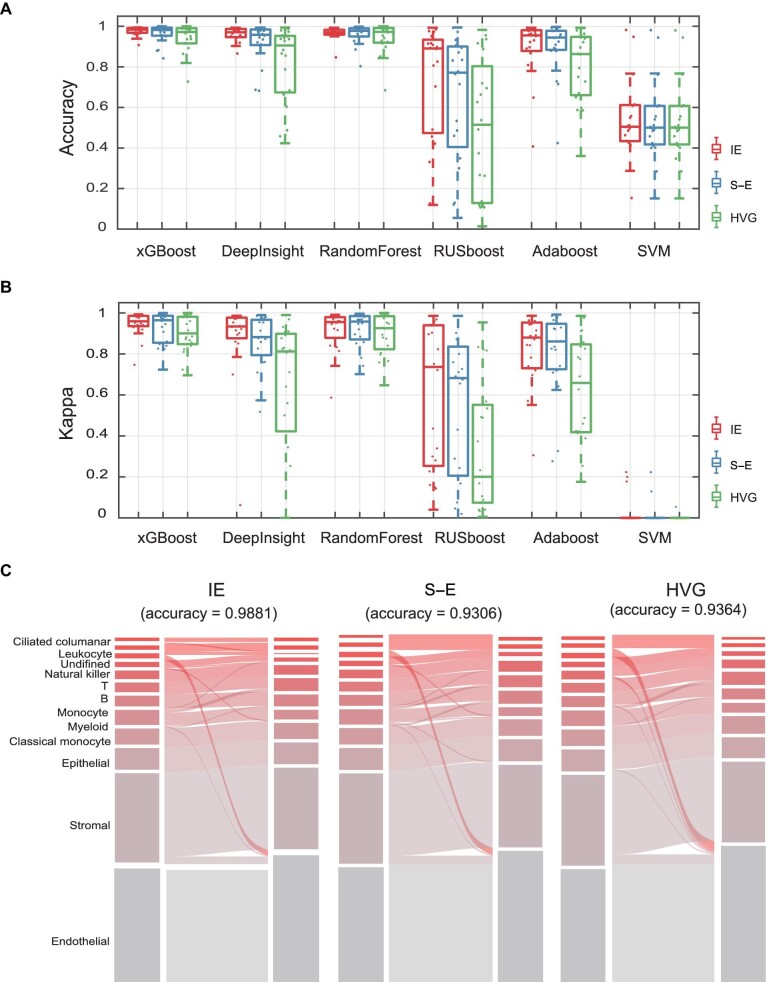
The performance of the IE model on cell type classification of the Tabula Muris dataset. (**A**) Classification accuracy was measured on 20 single-cell datasets by using six classification methods. The center line indicates the median classification accuracy. The lower and upper hinges represent the 25th and 75th percentiles, respectively. Each dot represents the accuracy for one dataset. (**B**) Kappa coefficient evaluation for 6 methods on 20 single-cell datasets from Tabula Muris. The center line indicates the median kappa coefficient. The lower and upper hinges represent the 25th and 75th percentiles, respectively. Each dot represents the mean kappa coefficient of one dataset by using 10-fold cross-validation. (**C**) Sankey plots show the xGBoost classification results of the Large-Intestine dataset based on genes selected by IE, S–E, and HVG.

Next, we considered six state-of-the-art machine learning classifiers: xGBoost (Chen and Guestrin, 2016), DeepInsight (Sharma et al., 2019), RandomForest (Breiman, 2001), Rusboost (Seiffert et al., 2008), Adaboost (Freund and Schapire, 1997), and SVM (Fan et al., 2005). The Tabula Muris dataset consisting of cells from 20 organs was used to evaluate the IE model and two other methods (S–E and HVG). As shown in Figure 3A, the IE model performed better than other feature selection methods in all the datasets, with only a few exceptions. Furthermore, the kappa coefficient was calculated to quantify the accuracy of the six classification methods based on different feature selection methods by performing 10-fold cross validation (Figure 3B). The Sankey results (Figure 3C) show the performance of these three feature selection methods on Brain Non-myeloid data by using xGBoost. The accuracies were 0.9881 for IE, 0.9306 for S–E, and 0.9364 for HVG. Clearly, the IE model (high-IE genes) significantly improved the accuracy of these classification methods (Figure 3A and B).

### Case study on single-cell datasets with dropout cells

We applied the IE model to the Tabula Muris dataset to cluster all the annotated cells and previously unassigned cells by Seurat (Stuart et al., 2019). For annotated cells, we demonstrated that the IE model can acquire high accuracy in classification (Table 1). For dropped cells, we validated that IE-guided clustering analysis can accurately classify these cells (Table 1).

**Table 1:** Summary of Tabula Muris dataset and the clustering results (ARI and the number of cell types) based on genes selected by the IE model.

Organ	Number of cells	Dropped cells	Number of annotated cell types	ARI	Number of annotated cell types (IE)	All cell types (IE)
Aorta	1113	706	4	0.5653	5	6
Bladder	1639	261	2	0.9797	2	3
Brain myeloid	4763	285	4	0.0228	5	7
Brian non-myeloid	5800	751	11	0.8089	6	11
Diaphragm	952	82	5	0.9851	5	6
Fat	5863	896	7	0.94	5	8
Heart	6002	1637	8	0.7791	5	9
Kidney	866	347	5	0.0103	6	6
Large intestine	4150	212	5	0.548	6	6
Limb muscle	1152	62	6	0.9545	5	7
Liver	982	268	6	0.4945	6	6
Lung	1924	208	13	0.7597	5	13
Mammary gland	2664	259	4	0.8723	5	5
Marrow	5356	319	22	0.6309	11	23
Pancreas	1962	398	9	0.6006	7	10
Skin	2465	155	5	0.4596	4	6
Spleen	1719	22	3	0.8992	3	4
Thymus	1581	232	3	0.0626	4	4
Tongue	1433	17	2	0.5534	3	3
Trachea	1392	42	4	0.9192	6	4

In addition, we demonstrated the application of the IE model in analyzing the Large-Intestine dataset (Tabula Muris Consortium et al., 2018), which includes 4150 annotated cells belonging to 5 cell types and 212 unassigned cells. Supplementary Figure S3 shows the selected high-IE genes for downstream clustering analysis. The marker or feature genes selected by Seurat had high PIE (a statistic to measure the total IE of markers) values on five cell types (Supplementary Figure S3B). In contrast, we identified six refined cell subtypes, each with its specific high-IE marker genes, by applying our IE model to the annotated cells of the Large-Intestine dataset (Supplementary Figure S3D). To investigate potential functions of these subtypes, we found considerable phenotypic diversity by comparing pathway activities. For example, epithelial cell was clustered into Cluster 2 and Cluster 3. Cluster 2 showed a strong correlation with the metabolic process, while the biosynthetic process was activated in Cluster 3. These results illustrated that high-IE genes as feature genes play an important role in clustering and classifying subpopulations of cells. To investigate whether these high-IE genes are effective in evaluating cell types, we studied the Large-Intestine dataset with dropped cells, which were unassigned in previous research. The clustering results suggested that the cluster number was seven. In other words, there may be another cell type by considering these dropped cells. We also found that some parts of the dropped cells were clustered well in Cluster 1 and Cluster 2 (Supplementary Figure S3F). Together with these results, we conclude that the IE model is able to identify new cell types with high accuracy and biological significance.

### The IE model identifies distinct subtypes in myeloid cells

**Figure 4:**
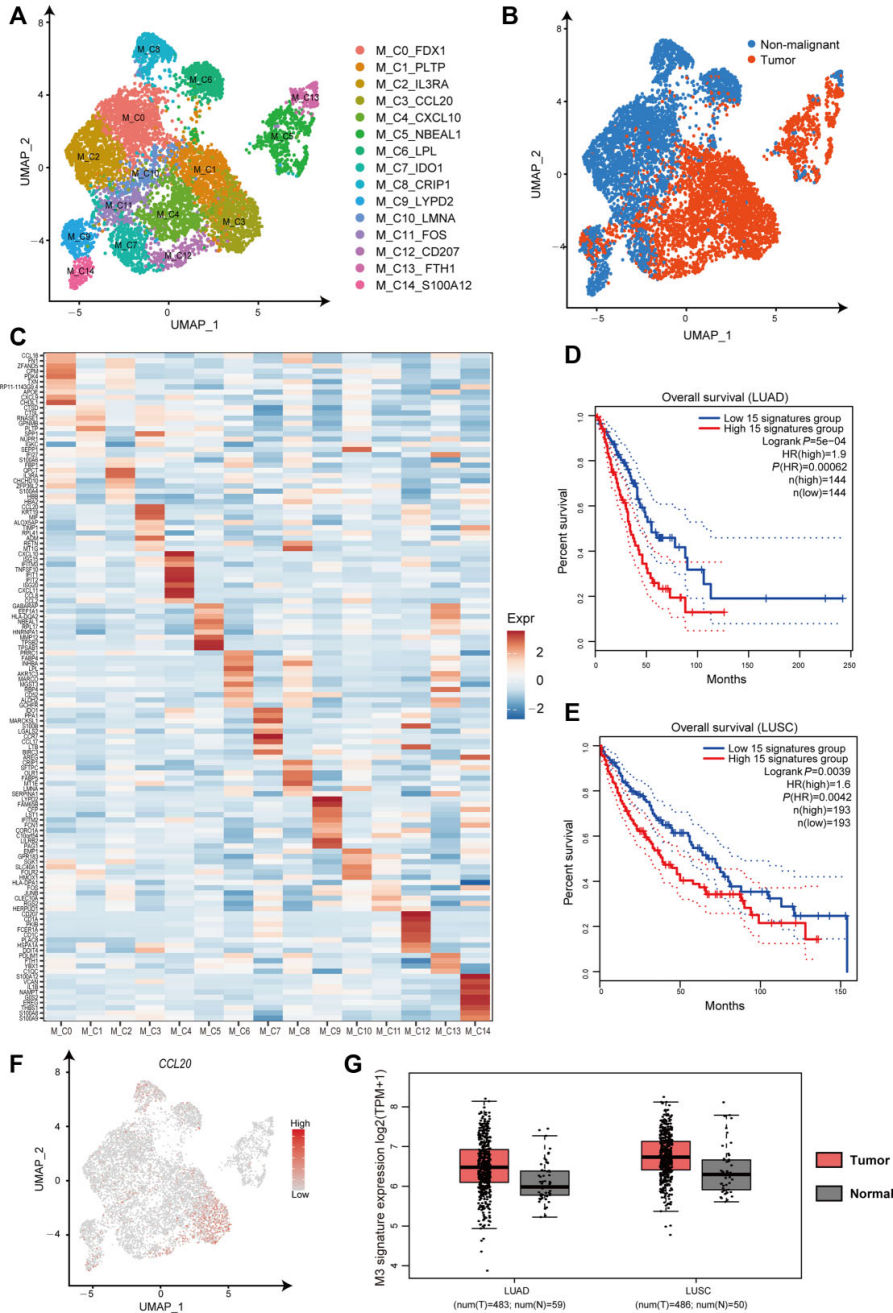
IE-guided cluster analysis identifies distinct subtypes in myeloid cells. (**A**) UMAP plots show the reclustered results of lung cancer-associated myeloid cells, colored by clusters. (**B**) UMAP plots of myeloid cells. Each cell is colored by its origin (tumor or nonmalignant lung). (**C**) The heatmap shows the relative expression levels of the top 10 marker genes (rows) in each cluster (columns). (**D** and **E**) Kaplan‒Meier survival analysis curves of TCGA LUAD (**D**) and LUSC (**E**) patients grouped by the top 15 markers of M_C3_CCL20. (**F**) The t-SNE plot shows the expression level of the *CCL20* gene. (**G**) The subtype M_C3_CCL20 (M3) signature expression levels in tumor and normal samples of LUAD and LUSC patients.

Tumor-associated macrophages (TAMs) are important components of the tumor microenvironment, which regulate key steps in tumor development and are significantly correlated with prognosis (Lewis and Pollard, 2006). However, single-cell transcriptome has shown that TAMs in gliomas (Müller et al., 2017) and metastatic renal cell carcinoma (Kim et al., 2016) express markers of different activation states (M1/M2) simultaneously, suggesting that macrophage polarization is not a bipolar state but a continuous, dynamic process. Here, we demonstrated and re-analyzed a previously published myeloid cell dataset from human lung cancer by the IE model (Lambrechts et al., 2018). Fifteen distinct clusters were identified (Figure 4A; Supplementary Figure S4) and five clusters (M_C1_PLTP, M_C3_CCL20, M_C4_CXCL10, M_C5_NBEAL1, and M_C12_CD207) were abundant in tumor (Figure 4B). The signatures of the subtype M_C1_PLTP were significantly enriched in regulated exocytosis, activation of immune response, and leukocyte migration (Supplementary Figure S5). The chemokin gene, *CCL20*, which recruits regulatory T cells and largely contributes to the progression of a variety of cancers (Chen et al., 2020), was expressed in the subtype M_C3_CCL20 (Figure 4C). The cluster M_C4_CXCL10, characterized by several chemokin genes, i.e. *CXCL10, CCL8, CCL2*, and *CXCL11* (Figure 4C), represented M1-like exudative macrophages (Tighe et al., 2011). Moreover, pathway activity analysis showed a significant enrichment in virus response and interferon γ response (Supplementary Figure S5). The genes of M_C5_NBEAL1 were involved in translation initiation and antigen processing (Supplementary Figure S5). *CD207*, which is only expressed in Langerhans cells, was highly expressed in the cluster M_C12_CD207 (Supplementary Figures S4 and S6). Notably, patients highly expressing the marker genes of M_C3_CCL20 have remarkably worse overall survival in both The Cancer Genome Atlas (TCGA) lung adenocarcinoma (LUAD) cohort dataset and lung squamous cell carcinoma (LUSC) cohort dataset (Figure 4D and E). The expression levels of both *CCL20* gene and the signatures of M_C3_CCL20 were higher in tumor (Figure 4F and G). Thus, the biological function of M_C3_CCL20 deserves further investigation.

## Discussion

scRNA-seq enables us to analyze the transcriptome on individual cells. By scRNA-seq technology, many genes can be detected at a single-cell resolution; however, only a small number of informative genes can reflect biological variability in a specific cellular process. It is usually a challenge to identify those informative genes due to noisy data and the dropout problem. Feature selection can reduce the technical noise of data and enhance the computional effectiveness in terms of accuracy and cost, i.e. for clustering or pseudo-time trajectory inference. Here, we construct the IE model to solve the feature selection problem by extracting the EE from the TE of each gene based on scRNA-seq data. In other words, we show that the TE of a gene can be theoretically decomposed into the IE and the EE of this gene, respectively. The IE model can accurately capture the informative genes or high-IE genes, thus enhancing the computational efficiency of scRNA-seq analysis. These results also imply that the IE or intrinsic fluctuation/variability of a gene serves as information rather than noise at gene expression level for cell clustering/classification or gene regulation, in contrast to its total entropy/fluctuation. At the cellular level, we notice the recent work on the role of cell-to-cell variability in cellular information transmission (Wada et al., 2020), which shows that cell-to-cell variability serves as information but not noise by defining intracellular variability and extracellular variability.

In this work, we first present the high sensitivity and good performance of high-IE genes for clustering analysis in both simulated datasets and real datasets. Then, by applying different clustering methods and classification methods on the high-IE genes, we also show their highly stable and accurate performance.

The IE model can be broadly applied to many downstream analyses of scRNA-seq data. Here, we showed that high-IE genes successfully identified cell subpopulations with high accuracy and biological significance on the Large-Intestine dataset. We also found a novel macrophage subtype M_C3_CCL20 in lung cancer by IE-guided analysis, and cells of M_C3_CCL20 were related with worse prognostic outcomes in lung cancer (LUAD and LUSC). Overall, the IE model can not only capture the informative genes or high-IE genes by eliminating their ‘noisy’ fluctuations based on data, but also improve the accuracy and stability of scRNA-seq analysis. As a future work, by exploiting the network features or dynamical features of the informative genes or high-IE genes, the IE model can be applied to the identification of network biomarkers (Liu et al., 2016; Zhao et al., 2016; Dai et al., 2019; Lu et al., 2019; Li et al., 2020; Zhang et al., 2021) for disease diagnosis/prognosis and further for the detection of dynamic network biomarkers (Chen et al., 2012; Li et al., 2017; Liu et al., 2018; Yang et al., 2018; Jiang et al., 2020; Shi et al., 2021) for disease prediction.

## Materials and methods

### IE model

The fluctuation of a random variable in a complex system is considered to result from the intrinsic randomness generated by its inherent dynamics and the extrinsic randomness influenced by its environment (Hilfinger and Paulsson, 2011). The entropy can capture the degree of fluctuation. Theoretically, the TE of a gene *x* can be decomposed into IE and EE, i.e. $E_{{\rm{tot}}}^x = \ \ E_{{\rm{int}}}^x + \ E_{{\rm{ext}}}^x$.

The IE of a gene results from its inherent dynamics. The EE of this gene represents the ‘noisy’ fluctuation/effect caused by all other genes, i.e. summation of random interacting effects from all other genes to gene *x*, though the interaction of two genes reflects the biological information.

Here, we assume that the log-transformed normalized expression data of genes follow the normal distribution. We applied the entropy to estimate the degree of fluctuation of each gene from the gene expression data. The TE of a random variable or gene *x* can be defined as

(1), \begin{equation*}E_{{\rm{tot}}}^x = \ - \mathop \int \nolimits_{ - \infty }^{ + \infty } p\left( x \right)\ \ln p\left( x \right){{d}}x\end{equation*}

where $p( x )$ is the probability distribution function of gene *x.*

Let the number of total genes be *m + 1*. TE represents the total fluctuation of gene *x*, including internal and external effects. Actually, other *m* genes, i.e. $Z = ( {{z_1},\ {z_2},\ \cdots ,\ {z_m}} )$, can have an effect on gene *x*, and such an effect is defined as the EE of gene *x*. Thus, theoretically we can easily show that the TE ($E_{{\rm{tot}}}^x$) of gene *x* is decomposed as the IE ($\ E_{{\rm{int}}}^x$) and the EE ($\ E_{{\rm{ext}}}^x$) of this gene, i.e.

(2). \begin{equation*}E_{{\rm{tot}}}^x = \ \ E_{{\rm{int}}}^x + \ E_{{\rm{ext}}}^x\end{equation*}

To show Equation (2), specifically, mutual information (MI) can be used to evaluate the association between two genes and further derive this decomposition. In our model, we apply MI to evaluate the effect of all other genes *Z* on gene *x*, which is actually the EE of gene *x* and defined as

(3), \begin{equation*}\ E_{{\rm{ext}}}^x = {\rm{\ MI}}\left( {x,{\rm{\ }}Z} \right) = {\rm{\ }}\int\!\!\!\int p\left( {x,Z} \right){\rm{ln}}\frac{{p(x,{\rm{Z}})}}{{p\left( x \right)p({\rm{Z}})}}{{d}}x{{d}}Z\end{equation*}

where $p( {x,\ Z} )$ is the joint probability distribution function of *x* and *Z*; ${p_i}( x )$ and ${p_i}( Z )\ $are the probability distribution functions of *x* and *Z*, respectively.

Thus, the inherent fluctuation of gene *x* can be obtained by eliminating the EE from the TE of gene *x*. By substituting Equation (1) and Equation (3) into Equation (2), we can derive such fluctuation as the IE of gene *x* as

(4). \begin{eqnarray*} E_{\rm int}^x &=& E_{\rm tot}^x - \ E_{\rm ext}^x\nonumber\\ & =& - \int p\left( x \right)\ \ln p\left( x \right)dx - \int\!\!\!\int p\left( {x,Z} \right){\rm{ln}}\frac{{\left. {p(x,{\rm{Z}}} \right)}}{{\left. {p\left( x \right)p({\rm{Z}}} \right)}}dxdZ\nonumber\\ &=& - \int\!\!\!\int p\left( {x,Z} \right){\rm{ln}}\left. {\! p(x|{\rm{Z}}} \right)dxdZ\nonumber\\ &=& - \int\!\!\!\int \left( {x,Z} \right){\rm{ln\ }}p\left( {x,Z} \right)dxdZ + \int\!\!\!\int p\left( {x,Z} \right)\ln p\left( Z \right)dxdZ\nonumber\\ &=& \ H\left( {x,Z} \right) - H\left( Z \right)\end{eqnarray*}

Then, we can estimate $E_{\rm int}^x$ of each gene *x* by probability distribution estimation methods based on Equation (4).

Here, we emphasize that the IE of gene *x* is essentially conditional entropy (CE), and the EE of gene *x* is MI. Actually, we use Shannon entropy to measure the total fluctuation of each gene, called TE in this work. As shown in Equation (2), the TE or $E_{{\rm{tot}}}^x$ of each gene *x* can be decomposed into MI ($E_{{\rm{ext}}}^x$) and CE ($E_{{\rm{int}}}^x$), i.e. TE(*x*) = MI(*x, Z*) + CE(*x*|*Z*), where $Z = ( {{z_1},\ {z_2},\ \cdots ,\ {z_m}} )$ is all other genes or all remaining genes with respect to this gene *x* in a dataset. MI(*x, Z*) is used to evaluate the association between this gene *x* and all remaining genes, i.e. focusing on this gene *x*, MI(*x, Z*) is actually the effect of all remaining genes on this specific gene *x*, and thus can be viewed as the external effect of all remaining genes on this specific gene *x*. On the other hand, CE(*x*|*Z*) is the CE of this specific gene *x* given all remaining genes, and thus can be viewed as the internal effect of this gene *x*. Therefore, we refer to CE(*x*|*Z*) = $E_{{\rm{int}}}^x$ and MI(*x, Z*) = $E_{{\rm{ext}}}^x$ as IE and EE with respect to this gene *x*, respectively.

As a special case for the multivariate normal distribution, Equation (4) can be simply expressed as

$$\begin{equation*}E_{{\rm{int}}}^x = \ \frac{1}{2}(\ln 2\pi + 1) + \ \frac{1}{2}{\rm{ln}}\frac{{\left| {{\Sigma _{x,\,Z}}} \right|}}{{\left| {{\Sigma _Z}} \right|}}\end{equation*}$$

due to $H( {x,\ Z} ) = \frac{{( {m + 1} )}}{2}(\ln 2\pi + 1) + \frac{1}{2}{\rm{ln}}| {{\Sigma _{x,\, Z}}} |$ and $H( Z ) = \frac{m}{2}(\ln 2\pi + 1) + \frac{1}{2}{\rm{ln}}| {{\Sigma _Z}} |$, where $( {x,\ Z} )\sim N( {{\mu _{x,\ Z}},{\rm{\ }}{\Sigma _{x,\, Z}}} )$ and $Z\sim N( {{\mu _Z},{\rm{\ }}{\Sigma _Z}} )$. µ and $\Sigma $ are the mean value and covariance matrix of a normal distribution, respectively. This approximation provides an efficient way to estimate the IE of each gene.

As shown in Supplementary Figure S1, we observed that the EE (or MI) between gene *x* and all other genes has a noisy effect. Actually, EE is the summation of random interactions (random variables) from all other genes to gene *x*, and thus can be viewed as the random fluctuation of the environment on this gene. In particular, we can show that EE actually approximately follows a Gaussian distribution if the number of genes is sufficiently large based on the central limit theorem. Thus, by eliminating this ‘noisy’ fluctuation or EE from TE, we can obtain the inherent fluctuation or IE of gene *x*, which reflects the inherent dynamics of gene *x* in a cellular process and thus provides rich information on scRNA-seq analysis.

Theoretically, all other genes, which may have interactions with gene *x*, should be considered in our IE model, but only a small number of genes have direct interactions with gene *x* in a real biological system. To reduce the complexity of computation to estimate the EE of each gene, we used principal component analysis (PCA) to reduce the dimension of the data, and the top PCs were used to approximately obtain the EE and further IE of each gene.

### Performance comparison in simulated datasets and real datasets

To demonstrate the performance of the IE model, we considered some popular feature selection methods, such as HVG (Brennecke et al., 2013) and the S–E model (Liu et al., 2020), to compare to our model. The HVG method identifies informative genes through distinguishing true biological variability from highly technical noise. The S–E model is an entropy-based feature selection method that can detect variable genes by hypothesis test. In addition, we further applied simulated datasets and real datasets to validate the performance of the IE model.

The Splatter (Zappia et al., 2017) package was applied to generate scRNA-seq count data to evaluate the performance of the feature selection methods. These datasets generated by Splatter follow a negative binomial. We simulated 600 datasets to test the performance of feature selection in clustering analysis. Each simulated dataset consisted of 500 cells of five groups with 20000 genes. The cell ratio of the five groups was set to 25:75:100:100:200. SIMLR (Wang et al., 2018) was applied to clustering analysis of the simulated datasets.

We also applied 14 public scRNA-seq datasets to assess the performance of the different feature selection methods in unsupervised clustering analysis (Supplementary Table S1). Datasets were sequenced with different protocols, containing Smart-seq2, 10x Genomics, CEL-seq2, and SMARTer protocol. We evaluated the performance of the three different feature selection methods in the context of clustering when selecting different numbers (500–4000) of genes. The Seurat (Stuart et al., 2019) package was used to perform clustering analysis. For droplet-based scRNA-seq datasets, the first 20 PCs were used for unsupervised clustering. For smart-seq2 scRNA-seq datasets, the first 10 PCs were used for unsupervised clustering.

### Evaluating the performance of the IE model by different clustering methods

To demonstrate the robustness of the IE model, we considered some widely used single-cell clustering methods such as SC3 (Kiselev et al., 2017), SIMLR (Wang et al., 2018), and Seurat (Stuart et al., 2019) to evaluate its performance. A smart-seq2 scRNA-seq dataset (Chu-time) and a droplet-based scRNA-seq dataset (Heart and Aorta) were considered for comparison. We selected the top 1000 genes by the IE model in each dataset, and SC3, SIMLR, and Seurat were used for clustering analysis. The true cell labels were defined in the original publication. ARI was used to evaluate the clustering results.

### Evaluating the performance of the IE model by different classification methods

*Dataset preparation.* To evaluate the performance of different feature selection methods in the context of various classification methods, we utilized one public single-cell dataset from Tabula Muris. We chose FACS datasets with 20 organs for subsequent processing. The Tabula Muris mouse data (Tabula Muris Consortium et al., 2018) were downloaded from https://tabula-muris.ds.czbiohub.org/.

*Performance evaluation.* To illustrate the performance of the IE model in real datasets (Table 1), we performed cross-validation experiments using six classification methods: xGBoost (Chen and Guestrin, 2016), DeepInsight (Sharma et al., 2019), Random Forest (Breiman, 2001), RUSboost (Seiffert et al., 2008), Adaboost (Freund and Schapire, 1997), and SVM (Fan et al., 2005).

(i) For each organ dataset, we only used the annotated cells provided by the authors and then identified the top 2000 genes with different feature selection methods, respectively, for downstream classification. (ii) We then drew a training sample *Z* from one organ dataset *D*, uniformly at random without replacement of each cell type. By default, the size of *Z* was set to 3/4 that of *D*. (iii) We further trained different classifiers using the training dataset with the top 2000 genes selected by different methods. (iv) The remaining cells (1/4 by default) were used to calculate the accuracy score and Cohen's kappa coefficient. And 10-fold cross-validation was performed to calculate Cohen's kappa coefficient using the K-fold cross validation routine of R and Matlab.

### Case study on dropped cells

We applied the IE model to Large-Intestine data from Tabula Muris with 4150 annotated cells and 212 dropped cells. We used the original Seurat R package to identify the cluster marker (feature) genes. To check the performance of marker genes in the IE model, we introduced the statistic PIE to measure the total IE of markers as

$$\begin{equation*}{\rm{PIE}} = \ \frac{{\mathop \sum \nolimits_i^k E_{{\rm{int}}}^m}}{{\mathop \sum \nolimits_i^k E_{{\rm{int}}}^t}},\end{equation*}$$

where *m* represents the number of marker genes among cells and *t* denotes the number of top IE genes. We set the default value of *k* to 10. The clustering results were visualized in 2D projection of UMAP with default parameters.

### Analysis of myeloid-like cells

To verify the ability of the IE model to identify novel subtypes, we performed IE-guided clustering analysis on myeloid cells of human lung cancer (Lambrechts et al., 2018). First, we excluded genes expressed in <10 cells and filtered out cells expressing <600 genes. Consequently, 14750 genes and 8172 cells were applied for futher analysis. Then, we used the IE model or method on the normalized data to select the informative genes. The top 2000 high-IE genes were applied for downstream analysis using Seurat (resolution = 0.8). Finally, we obtained 15 myeloid cell subtypes and UMAP was used for visualization. Furthermore, we used all markers of each cluster (log-foldchange >0.25) to perform Gene Ontology functional enrichment analysis using Metascape (Zhou et al., 2019) with default parameters (min overlap = 3, *P*-value cutoff = 0.01, min enrichment = 1.5) and g:Profiler (Raudvere et al., 2019). We performed survival analysis and expression boxplot using GEPIA2 (Tang et al., 2019) with the top 15 differential genes, which had the minimal *P*-value and log-foldchange >0.5.

### Data availability

All the datasets used in this study were obtained from their public accessions, and detailed information can be found in Supplementary Table S1 and the References. All the scripts in this study are available in the GitHub repository: https://github.com/LinLi-0909/IEntropy.

## Supplementary Material

mjac008_Supplemental_File

## Notes

**Author contributions:** L.L. and L.C. conceived and designed this work. L.L., H.T., R.X., H.D., and R.L. acquired, analyzed, and interpreted the data. L.L. and H.T. wrote the source code used in this work. L.L. and H.T. drafted the manuscript. L.C. revised the manuscript.
