# Supplementary material for: Intrinsic entropy model for feature selection of scRNA-seq data
Source: J Mol Cell Biol. 2022 Jan 31;14(2):mjac008. doi: 10.1093/jmcb/mjac008 (PMC9175189; doi:10.1093/jmcb/mjac008)
Supplement: mjac008_Supplemental_File [file mjac008_supplemental_file.pdf]

Supplemental Material for

Intrinsic entropy model for feature selection of scRNA-seq data

Lin Li[1,2,#], Hui Tang[3,#], Rui Xia[1,2], Hao Dai[1], Rui Liu[3] and Luonan Chen[1,4,5,6 *]

[1]Key Laboratory of Systems Biology, Shanghai Institute of Biochemistry and Cell Biology, CAS Center for Excellence in Molecular Cell Science, Chinese Academy of Sciences, Shanghai 200031, China

[2]University of Chinese Academy of Sciences, Beijing 100049, China.

[3]School of Mathematics, South China University of Technology, Guangzhou 510640, China

[4]Center for Excellence in Animal Evolution and Genetics, Chinese Academy of Sciences, Kunming 650223, China

[5]Key Laboratory of Systems Biology, Hangzhou Institute for Advanced Study, University of Chinese Academy of Sciences, Chinese Academy of Sciences, Hangzhou 310024, China

[6]School of Life Science and Technology, ShanghaiTech University, Shanghai 201210, China

[#] These authors contributed equally to this work.

*Address correspondence to this author, Institute of Biochemistry and Cell Biology, Chinese Academy of Sciences, Yueyang Road 320, Shanghai 200031, China; Tel: +86-13391401383;

Email: lnchen@sibs.ac.cn

Luonan Chen, Ph.D.

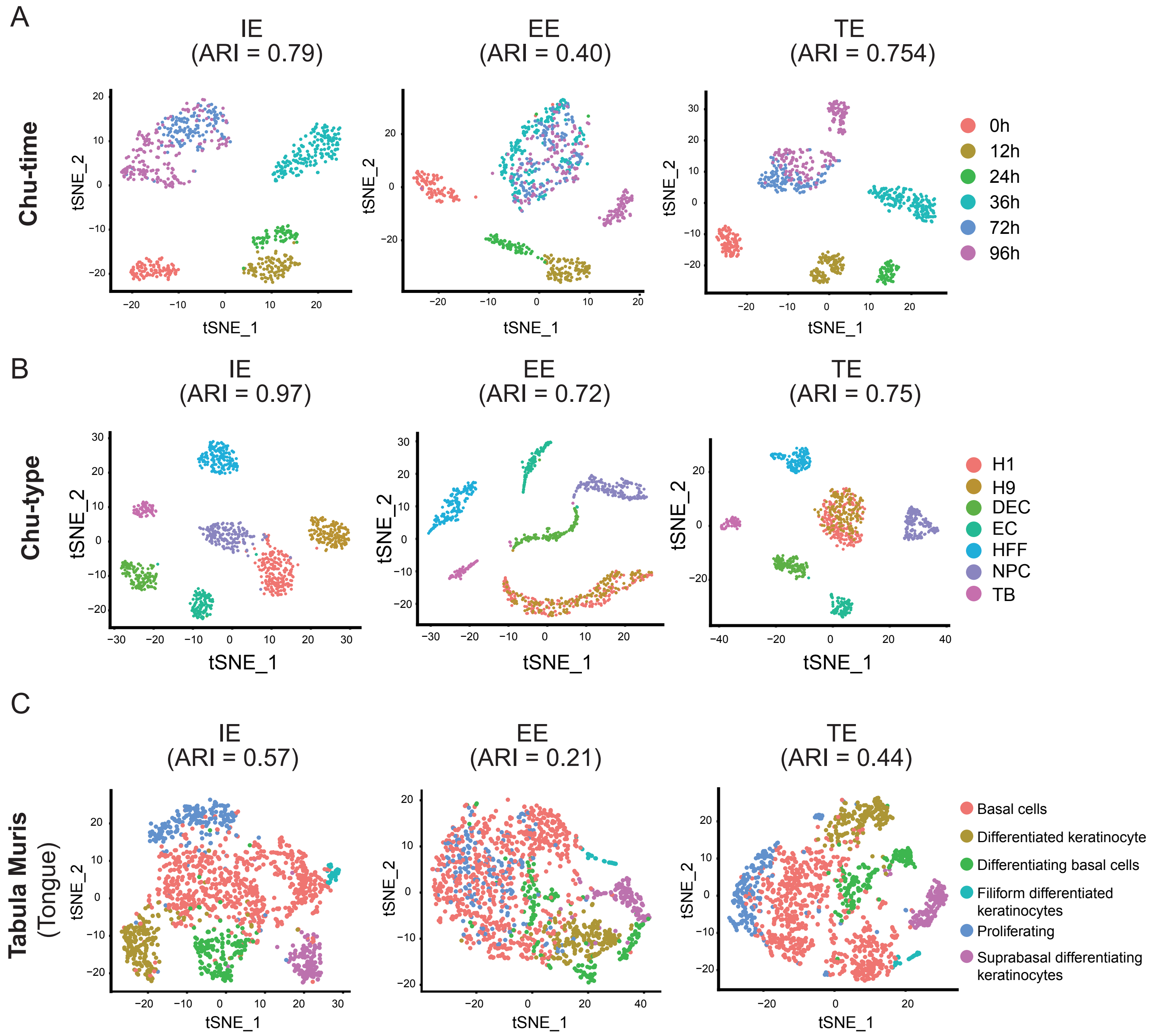

Figure S1. The performance of visualization and clustering in high-IE genes, high-TE genes, and high-EE genes. (A-C) The t-SNE plots show performance of dimension reduction and clustering by the genes of high IE, high TE, and high EE. The number of selected genes n = 100 for Chu-time dataset, n = 200 for Chu-type dataset, and n = 100 for Tabula Muris (Tongue). ARI is used to evaluate the clustering results.

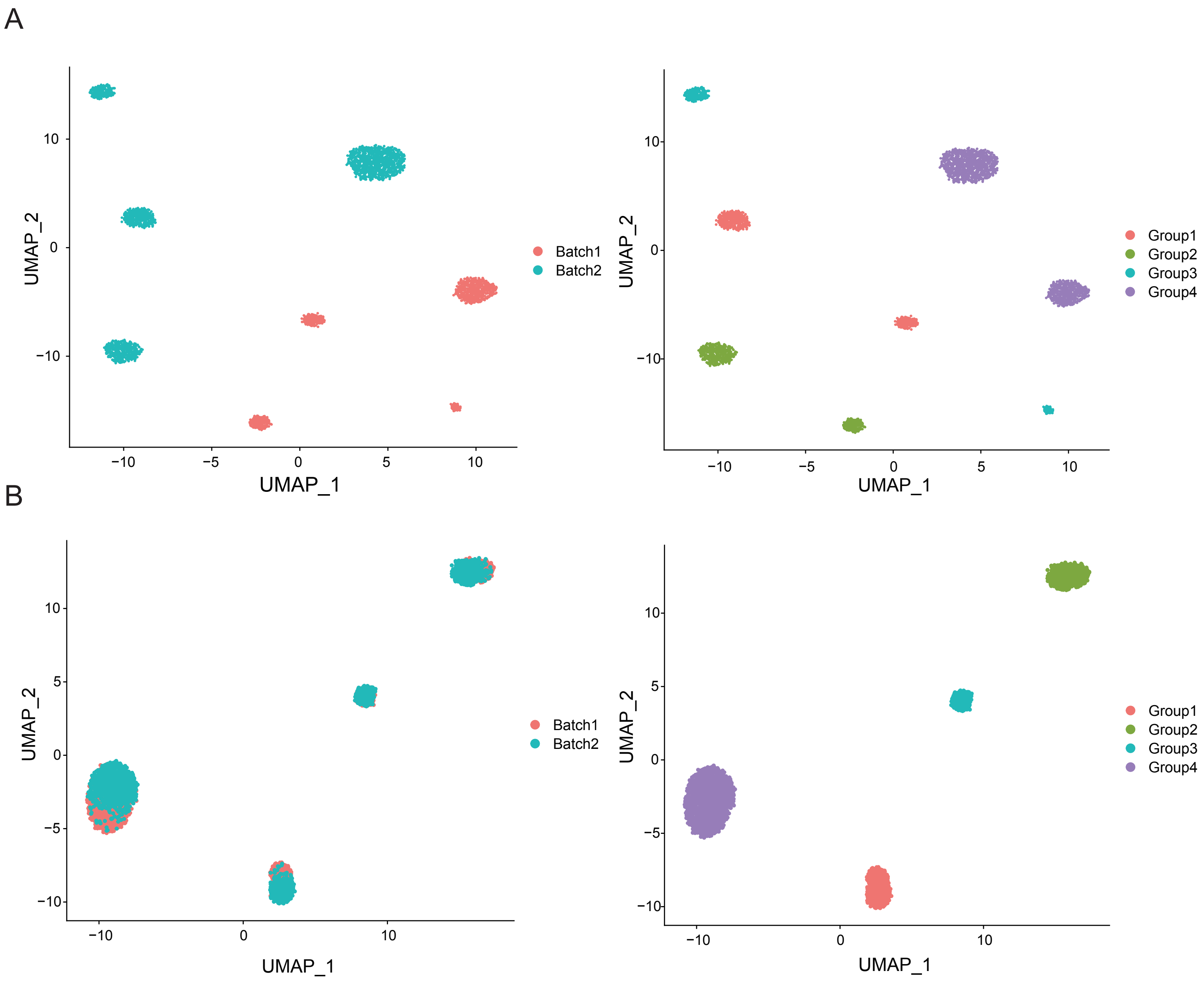

Figure S2. UMAP plots shown the simulated scRNA-seq data containing two batches of different cell types. (A) Data before correction (B) Data after correction based on our IE model.

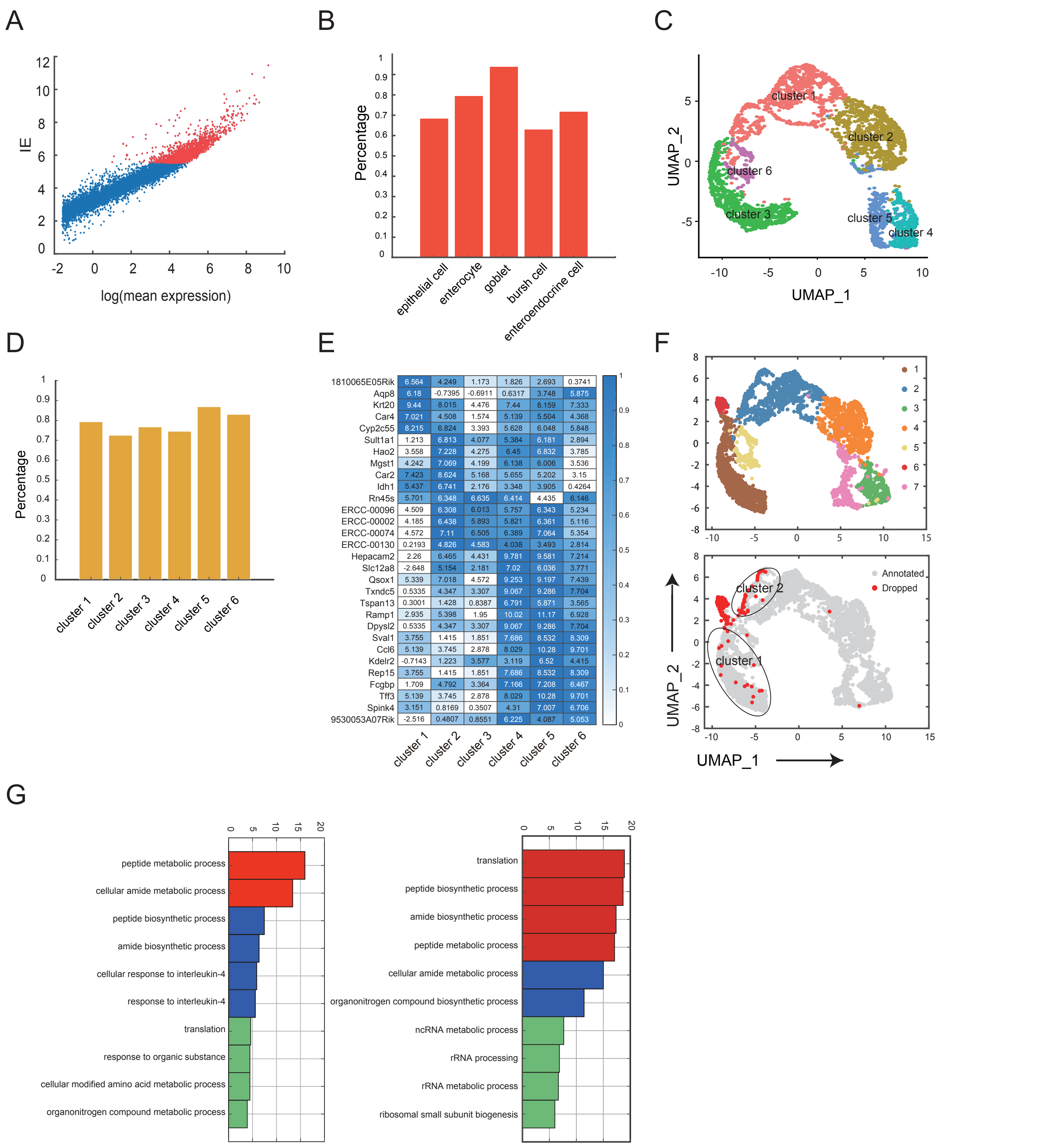

Figure S3. The application of IE model on Large-Intestine dataset. (A) The IE plot of large intestine dataset. Each dot represents a gene and red dots represent the top 2000 high IE genes for down-stream ananlysis. **(B)** the PIE values on five original cell types. **(C)** UMAP plot of annotated large intestine cells by Seuart. Each cell was colored by its origin labels. **(D)** the PIE values on six identified cell types. **(E)** Expression heatmap of cell-type specific genes of six clusters. **(F)** UMAP plot of all large intestine cells by Seurat(upper). UMAP plot shows the clustering result of dropped cells(down), red dots represent the drop cells and gray dots represent annotated cells. **(G)** Enriched pathways for cluster 1 and cluster 2.

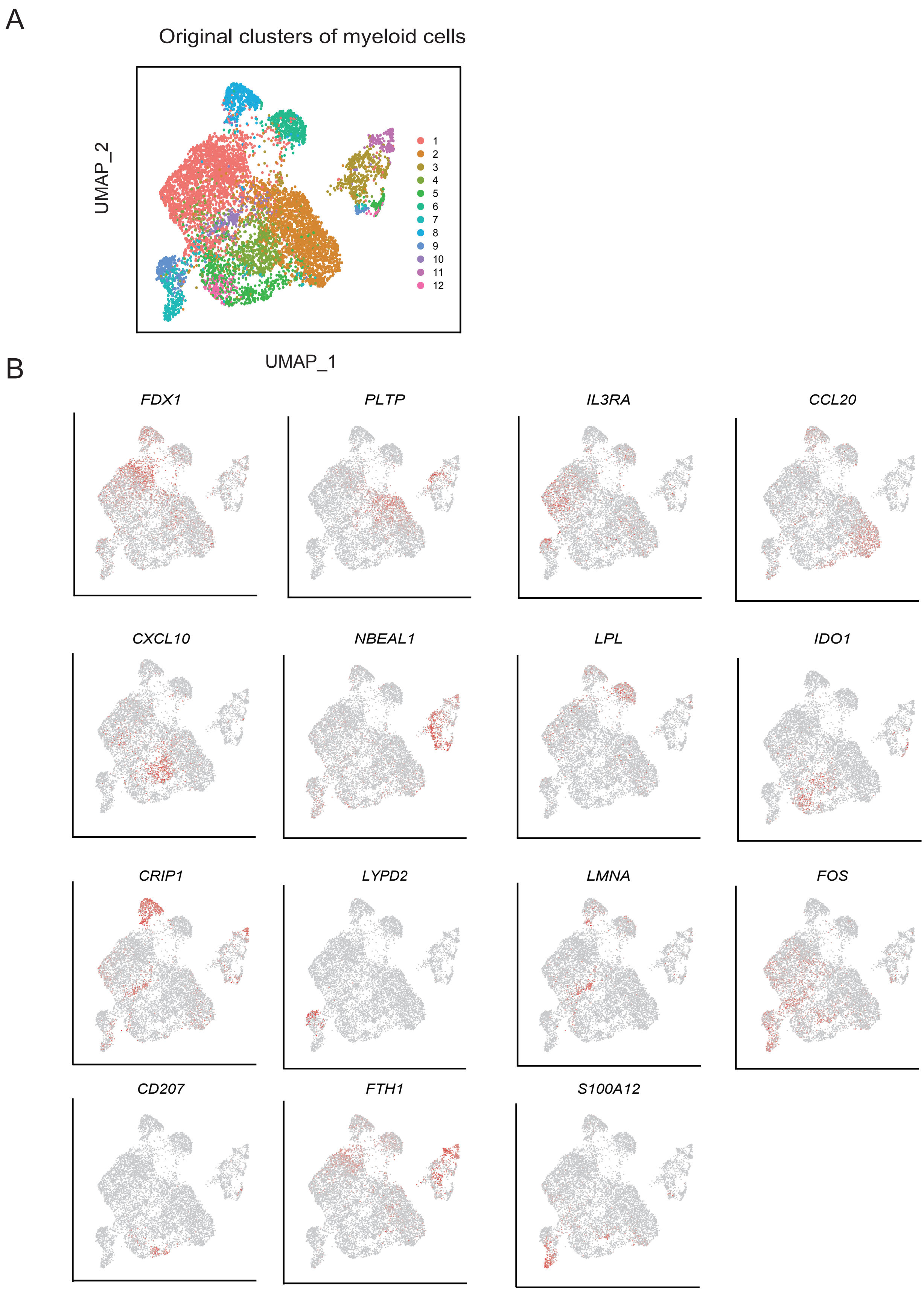

Figure S4. Cluster information of myeloid cells in original paper and expression levels of signature genes in re-clustered subtypes. (A) The cluster name of myeloid cells descripted in original paper. **(B)** t-SNE plots show the expression levels of signatures of re-clustered myeloid subtypes.

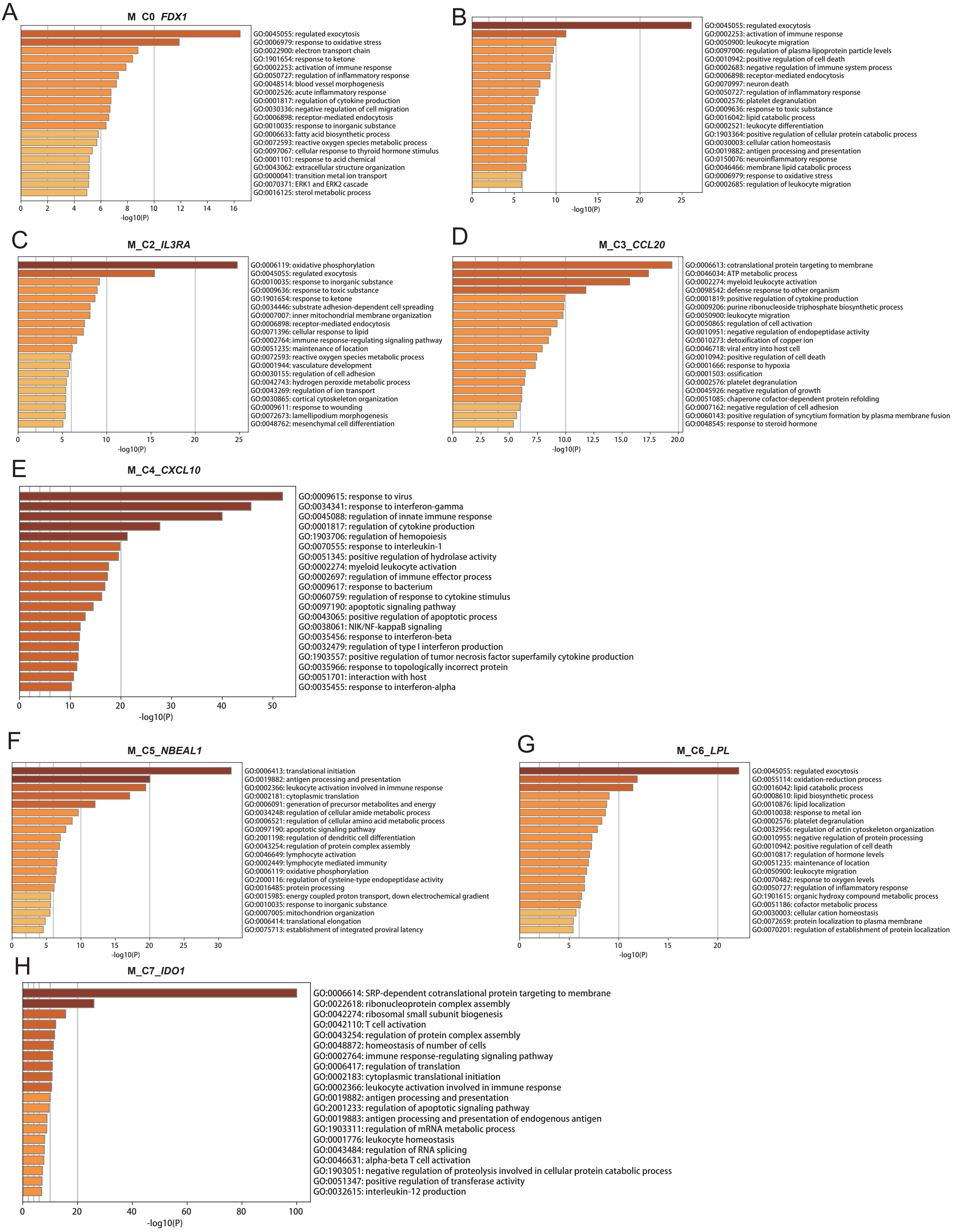

Figure S5. Significantly enriched gene ontology terms in subtypes of myeloid cells. (A-H) The bar plots show the significantly enriched gene ontology terms in subtype M0-M7.

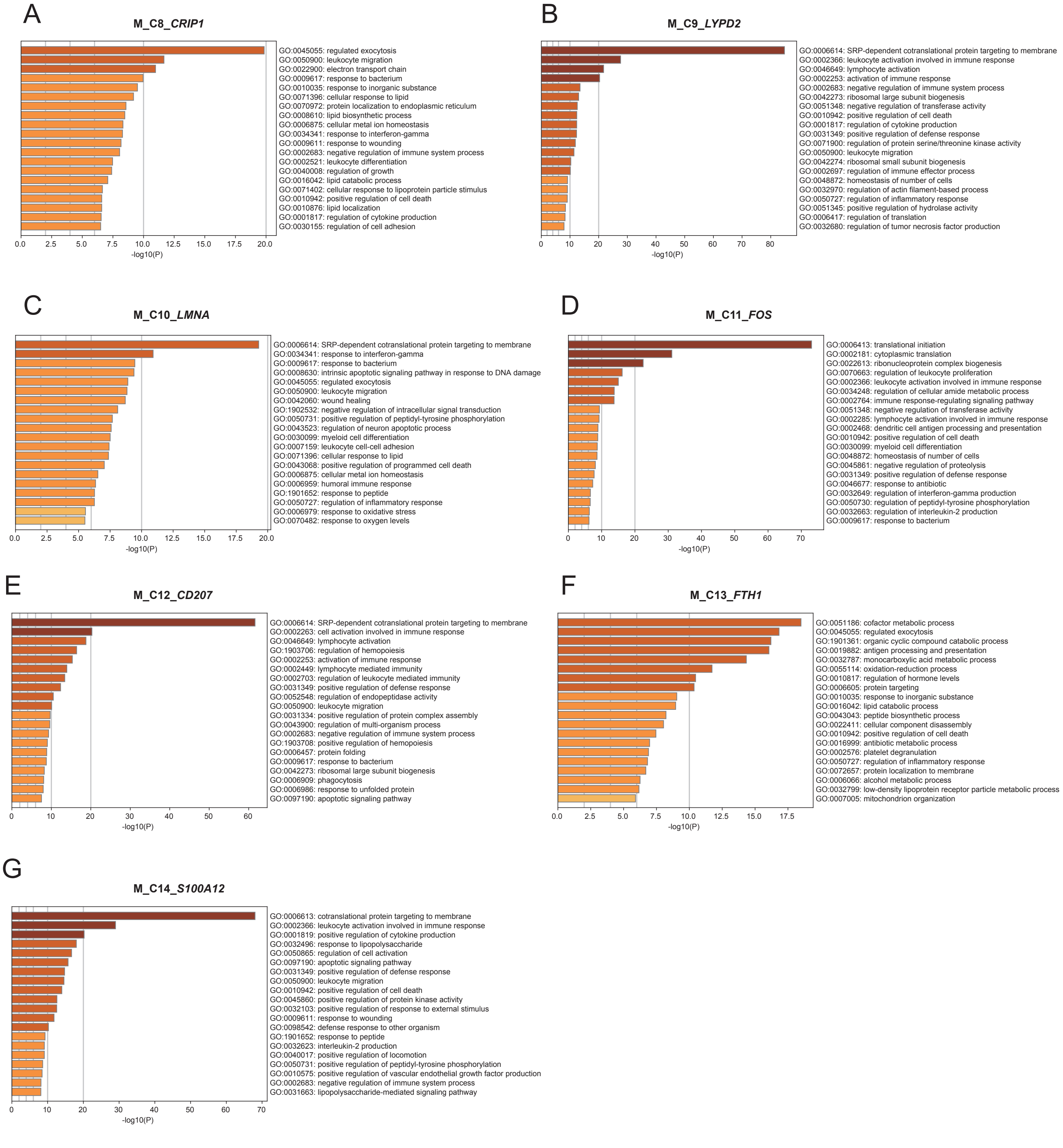

Figure S6. Significantly enriched gene ontology terms in subtypes of myeloid cells. (A-G) The bar plots show the significantly enriched gene ontology terms in subtype M8-M14.
